# The Prognostic Value of De Ritis Ratio on the Survival Rate of ICU-Admitted COVID-19 Patients During the Second Wave: A Retrospective Study in a Tertiary Care Hospital in India

**DOI:** 10.7759/cureus.52484

**Published:** 2024-01-18

**Authors:** Khushboo Raj, Bandana Kumari, Sushil Kumar, Akash Bansal, Palani Selvam Mohanraj, Soma Dey, Aishwarya Thakur

**Affiliations:** 1 Biochemistry, All India Institute of Medical Sciences, Patna, IND; 2 Biochemistry, All India Institute of Medical Sciences, Gorakhpur, IND

**Keywords:** critical care management, icu, diagnostic accuracy, survival rate, de ritis ratio, covid-19

## Abstract

Background

Prognostic markers are essential for optimizing COVID-19 patient care. This retrospective study examines the prognostic value of the De Ritis ratio (DRR) in intensive care unit (ICU)-admitted patients during the second wave of the pandemic.

Methods

A retrospective study of four-month duration (March to June 2021) was conducted on 161 ICU-admitted COVID-19 patients in a tertiary care hospital in India. The data included demographics, comorbidities, laboratory results, ICU admission dates, and survival outcomes. The De Ritis ratio was calculated on day 0, day 2, and day 5. The analyses included descriptive statistics, diagnostic accuracy, and logistic regression.

Results

Survival rates decreased with ICU stay: day 0 (survival, 58.4%; mortality, 41.6%), day 2 (survival, 54.5%; mortality, 45.5%), and day 5 (survival, 49.5%; mortality, 50.5%). De Ritis ratio's diagnostic accuracy varied, with increasing specificity and negative predictive value (NPV). Logistic regression showed higher day 5 De Ritis ratios, and male gender was associated with reduced survival odds.

Conclusion

The De Ritis ratio demonstrates promise as an early prognostic marker for COVID-19 patients, with an increase in predictive accuracy over time. The results emphasize the De Ritis ratio's potential as an early indicator of disease severity, offering clinicians a tool to recognize patients at higher risk and enhance the effectiveness of critical care interventions.

## Introduction

The global challenge posed by the severe acute respiratory syndrome coronavirus 2 (SARS-CoV-2), known as COVID-19, has placed immense pressure on healthcare systems worldwide [1]. As successive waves of infection have emerged, understanding the determinants of patient outcomes has become of paramount importance.

Originating from the work of Fernando De Ritis in 1957, the De Ritis ratio (DRR) signifies the ratio of serum activities of aspartate aminotransferase (AST) and alanine aminotransferase (ALT) in serum [2]. DRR serves as an indicator of hepatic injury and has historically been employed to assess liver-related conditions [3].

Elevated ALT levels indicate liver dysfunction, predominantly localized in hepatocyte cytosol, while increased AST levels suggest systemic involvement due to its presence in the cytosol and mitochondria of various organs including the heart, skeletal muscle, brain, and kidney, apart from the liver. Thus, the DRR has demonstrated its utility as a valuable marker not only for liver diseases but also for conditions affecting the heart and kidneys and even cancers [4]. DRR has proven itself to be a prognostic marker for various cancers such as bladder, renal cell, oropharyngeal, and pancreatic and in patients of cardiac arrest, acute myocardial infarction, ischemic stroke and those with SARS-CoV-2 viral infection [5,6].

Several studies have indicated that DRR values below 1.0 suggest moderate to severe liver damage, while ratios surpassing 1.0 indicate substantial liver impairment or progression to cirrhosis, particularly in cases of chronic viral hepatitis. The observation of notably elevated AST levels, sometimes reaching 20-50 times the normal range, has garnered attention in studies exploring COVID-19-related liver necrosis and virus-induced hepatitis [7].

In the context of COVID-19, emerging evidence suggests that the DRR could offer insights into disease severity and subsequent patient prognosis. DRR has also exhibited promise as a biochemical parameter for assessing outcomes in hospitalized COVID-19 patients [8]. However, limited data exist concerning the DRR within the subgroup of intensive care unit (ICU)-admitted patients who experienced fatal outcomes. Prognostic models are widely utilized in intensive care units for stratifying risks [9].

An in-depth analysis of morbidity and mortality among ICU-admitted COVID-19 patients, encompassing diverse variables including laboratory parameters and associated comorbidities, has been addressed in a previously published study by Kumari et al. (2023) [10].

COVID-19's second wave has presented unique challenges, marked by variations in disease presentation, virulence, and response to treatment strategies. The demand for critical care resources during this wave has necessitated effective triaging and management strategies to optimize patient outcomes. Amidst this backdrop, the potential of the DRR to serve as an early prognostic marker gains prominence. This retrospective study aims to investigate the prognostic value of the DRR at day 0, day 2, and day 5 of hospitalization in predicting the survival rate of patients admitted to the intensive care unit (ICU) during the second wave of COVID-19 in a tertiary care hospital in India.

By considering multiple time points, this study seeks to capture the evolving trajectory of the DRR and its association with patient survival. The evaluation of the DRR's diagnostic accuracy in predicting survival outcomes contributes to a deeper understanding of its utility in guiding clinical decision-making during a critical phase of patient care.

The insights derived from this study hold implications for patient risk stratification, resource allocation, and the development of personalized treatment strategies. Additionally, findings from this study could potentially inform the design of future interventions targeting patients at high risk of adverse outcomes, ultimately improving the management and prognosis of severe COVID-19 cases.

## Materials and methods

Study design

This study employed a retrospective observational design to assess the prognostic value of the DRR in predicting the survival rate of patients admitted to the ICU during the second wave of COVID-19 (from March 2021 to June 2021) in a tertiary care hospital in India. The study utilized data collected from electronic medical records of patients who were admitted to the ICU during this four-month period.

Study setting and population

The study was conducted at a tertiary care hospital located in the Bihar state of India. The study population comprised patients who were admitted to the ICU due to confirmed COVID-19 infection during the specified four-month duration.

Data collection

Patient data were retrospectively collected from electronic medical records. The data included demographic information, such as age, sex, and comorbidities, as well as laboratory test results, specifically the levels of aspartate aminotransferase (AST) and alanine aminotransferase (ALT). These biochemical markers were used to calculate the DRR. Additionally, the date of ICU admission and discharge, as well as survival outcomes, was recorded for each patient.

Inclusion and exclusion criteria

The inclusion criteria for patient selection were as follows: patients with a confirmed diagnosis of COVID-19 and patients who were admitted to the ICU during the specified four-month duration (March 2021 to June 2021). The exclusion criteria for patient selection were as follows: patients with missing AST/ALT data and patients with incomplete medical records.

DRR calculation

The DRR, defined as the ratio of AST to ALT levels, was calculated for each patient at three time points: day 0, day 2, and day 5 of hospitalization.

Statistical analysis

All statistical analyses were performed using Python version 3.7 (Python Software Foundation, Wilmington, DE). The patient information was entered in Microsoft Excel (Microsoft® Corp., Redmond, WA) for analysis. Categorical variables were presented as percentages, and the chi-square test was used to derive an association between two categorical variables. Continuous variables were presented as means ± standard deviations or medians with interquartile ranges, as appropriate. To evaluate the diagnostic accuracy of the DRR in predicting patient survival, the following measures were calculated: sensitivity, specificity, positive predictive value (PPV), negative predictive value (NPV), and receiver operating characteristic (ROC) curve. ROC curve was plotted for DRR on day 5 to obtain the area under the ROC curve (AUROC), cut-off score, and sensitivity of the cut-off score. A P-value of <0.05 was considered statistically significant.

Sample size justification

The sample size of 161 patients was determined based on the availability of data during the specified four-month study period. Further studies with larger sample sizes may further validate the findings of this study.

Data analysis plan

A pre-defined data analysis plan was formulated before conducting the statistical analyses to minimize potential bias and ensure transparency in the research process.

Data availability

Access to patient data was granted by the hospital authorities, ensuring compliance with data protection and privacy regulations.

## Results

**Figure 1:**
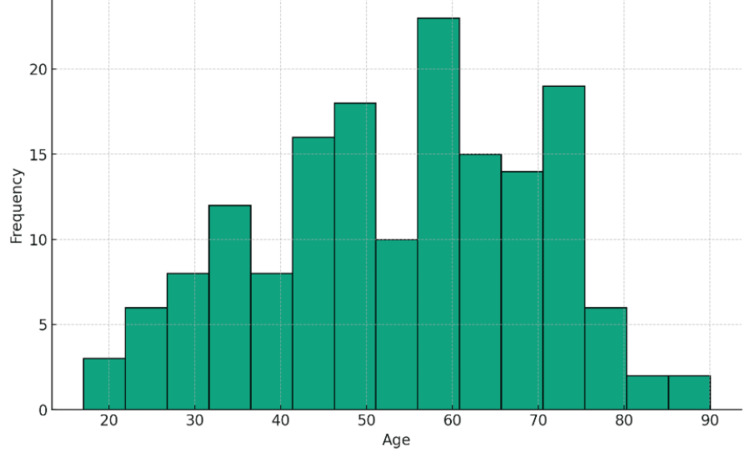
Age (years) distribution of ICU-admitted COVID-19 patients ICU: intensive care unit

A total of 161 patients were admitted to the ICU during the four-month study period. Demographic characteristics and the survival outcome of these patients are given in Table 1 and Figure 1. Among the 161 patients, 21 patients were discharged, and six patients succumbed within two days of ICU admission. The overall survival rate on day 1 was 94 (58.4%), and the overall mortality rate was 67 (41.6%). In the subsequent analysis, 134 patients were considered, excluding those who were discharged or died within the first two days of ICU admission. Among these patients, 18 were discharged, and five succumbed within three and four days of ICU admission. The overall survival rate on day 3 was 73 (54.5%), and the overall mortality rate was 61 (45.5%). The remaining 111 patients were followed up from day 5 until the date of discharge. Among these patients, 55 survived, and 56 succumbed during the follow-up period. The overall survival rate on day 5 and until complete follow-up was 55 (49.5%), and the overall mortality rate was 56 (50.5%).

**Table 1:** Gender distribution between survivors and non-survivors P-value < 0.05: statistically significant χ^2^, chi-square; df, degrees of freedom

Gender	Survival: n (%)	Non-survival: n (%)	Total	Test of significance
Male	56 (50.5%)	55 (49.5%)	111	χ^2^ = 9.62; df = 1; P-value = 0.002
Female	38 (76.0%)	12 (24.0%)	50	

These results indicate that the survival rates decreased as the duration of ICU admission increased. Notably, the overall mortality rate on day 5 and beyond surpassed the survival rate, highlighting the critical nature of this period for patient outcomes.

**Figure 2:**
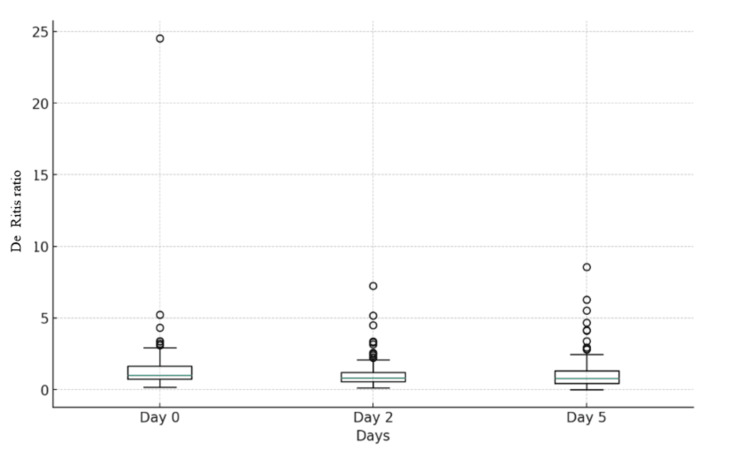
Box plot of De Ritis ratio on day 0, day 2, and day 5

**Figure 3:**
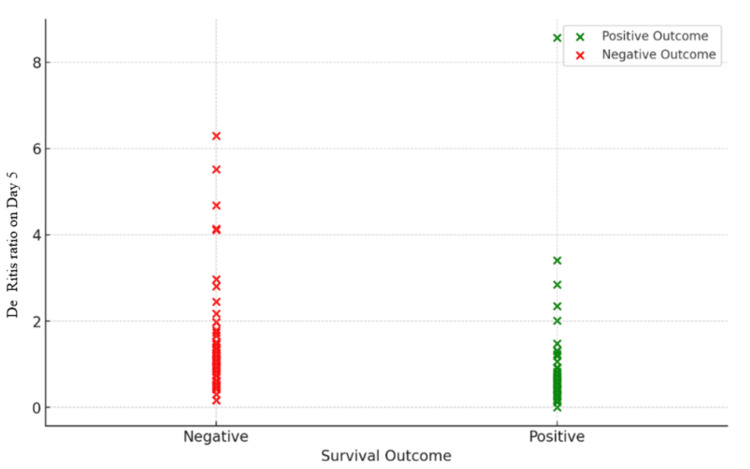
Survival outcome versus De Ritis ratio on day 5 Survival outcome denotes the total number of patients who survived (positive outcome) and who died (negative outcome) until day 5 of ICU admission ICU: intensive care unit

The subsequent sections will provide an in-depth analysis of the diagnostic accuracy of the DRR at different assessment days in predicting patient survival. The change in DRR over time at day 0, day 2, and day 5 was given in Table 2 and Figure 2. The survival outcome versus DRR on day 5 was highlighted in Figure 3. On day 0, the DRR displayed a high sensitivity of 98.94%, accurately identifying nearly all actual positive cases. However, its specificity was 0%, failing to correctly identify any of the true negative cases. The positive predictive value (PPV) stood at 57.76%, indicating the proportion of true positives among all positive predictions, while the negative predictive value (NPV) was 0%, denoting the proportion of true negatives among all negative predictions.

**Table 2:** AST, ALT, and De Ritis ratio at day 0, day 2, and day 5 P-value < 0.05: statistically significant. AST reference range: 0-37 IU/L. ALT reference range: 13-40 IU/L AST, aspartate aminotransferase; ALT, alanine aminotransferase

	Survivors (at the end of the four-month study period)	Non-survivors (at the end of the four-month study period)	
Observed values on day 0	n = 94	n = 67	P-value
AST (IU/L)	60.91 ± 57.93	93.64 ± 85.33	0.004
ALT (IU/L)	76.88 ± 98.56	88.08 ± 79.67	0.443
AST/ALT	1.36 ± 2.46	1.44 ± 0.98	0.801
Observed values on day 2	n = 73	n = 61	P-value
AST (IU/L)	54.26 ± 40.18	85.99 ± 88.17	0.006
ALT (IU/L)	84.97 ± 78.15	93.29 ± 101.00	0.592
AST/ALT	1.044 ± 1.05	1.13 ± 0.83	0.60
Observed values on day 5	n = 55	n = 56	P-value
AST (IU/L)	41.36 ± 29.85	74.33 ± 57.03	0.000
ALT (IU/L)	71.97 ± 67.35	98.36 ± 140.52	0.211
AST/ALT	Mean = 0.87 ± 1.22	Mean = 1.48 ± 1.25	0.010

Moving to day 2, the DRR demonstrated a sensitivity of 95.89%, effectively identifying a significant proportion of actual positive cases. Its specificity, however, was 4.92%, indicating a low ability to correctly identify true negative cases. The PPV was 54.69%, highlighting the proportion of true positives among all positive predictions, while the NPV was 50.00%, representing the proportion of true negatives among all negative predictions.

**Figure 4:**
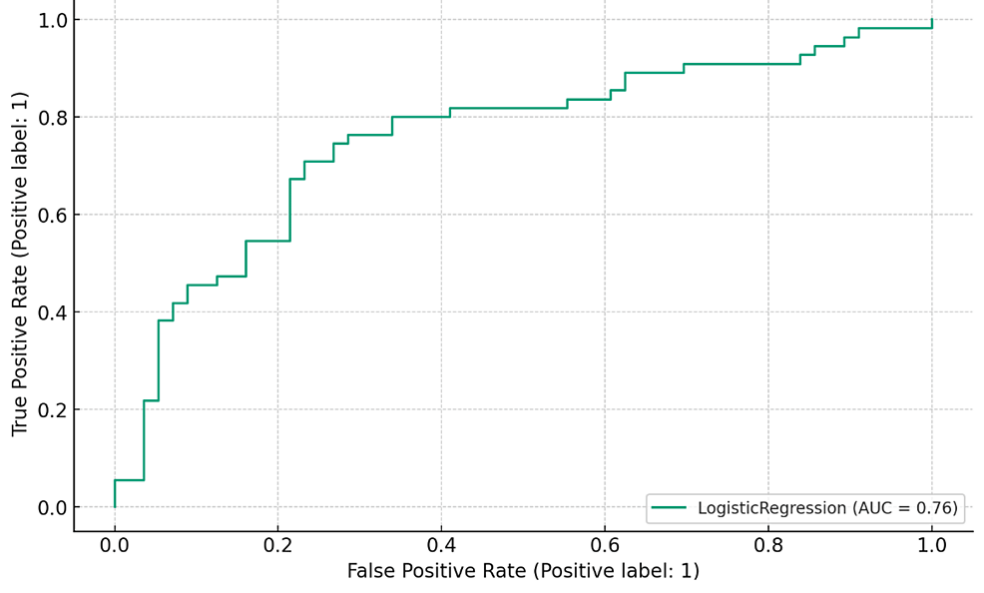
Receiver operating characteristic (ROC) curve for De Ritis ratio on day 5 AUC: area under the curve

On day 5, the DRR maintained a sensitivity of 81.82%, correctly identifying a substantial proportion of actual positive cases. Its specificity improved to 57.14%, indicating an increased ability to identify true negative cases. The PPV reached 65.22%, denoting the proportion of true positives among all positive predictions, while the NPV rose to 76.19%, representing the proportion of true negatives among all negative predictions. The De Ritis ratio exceeding 1 on the fifth day after ICU admission resulted in an area under the receiver operating characteristic (AUROC) of 0.76, with a confidence interval ranging from 0.665 to 0.849 (Figure 4).

Additionally, a logistic regression analysis was conducted to predict survival outcomes using gender and the DRR on day 5 as independent variables. The coefficient for gender was -1.1872, revealing that, while holding the DRR constant, males (encoded as 1) were associated with lower log odds of a positive survival outcome compared to females (encoded as 0). This implies that being male correlated with a reduced likelihood of survival within this model. Furthermore, the coefficient for the DRR on day 5 was -0.5574. This negative coefficient suggests that for every one-unit increase in the ratio, there was a corresponding decrease in the log odds of a positive survival outcome, keeping gender constant. This indicates that higher DRR on day 5 was linked to a decreased likelihood of survival.

The study highlights a decline in survival rates with prolonged ICU admission duration. The diagnostic accuracy of the DRR varied across assessment days. The logistic regression analysis underscored the association of being male and higher DRR on day 5 with a decreased likelihood of survival.

## Discussion

The present study aimed to assess the prognostic value of the DRR at different time points within the initial five days of ICU admission for COVID-19 patients during the second wave of the pandemic. By examining the association between DRR and patient survival, this study contributes valuable insights into the utility of a readily available biochemical marker in predicting clinical outcomes and informing critical care management strategies.

The DRR offers an intriguing promise in the clinical setting related to COVID-19 [11]. An assessment of its significance in relation to ICU-admitted COVID-19 patients, particularly during the pandemic's second wave, provides substantial insights into its potential utility as a biomarker for determining patient outcomes and adjusting critical care protocols.

One remarkable feature is the variation in the AST and ALT activity in various organs, with a distinct discrepancy in their clearance rates. This differentiation results in an equilibrium of aminotransferases in the serum and a DRR of <1 in healthy individuals [12]. Liver dysfunction is implied by a rise in ALT, given its presence mainly in the liver's cytoplasm, whereas AST can be found in multiple organs including the liver's cytoplasm and mitochondria [13]. The substantial surge in AST in non-survivors possibly signals systemic involvement.

The dynamic nature of the DRR's predictive power is evident in the temporal variations observed across different assessment days. Our findings reveal a notable decrease in survival rates as the duration of ICU admission increases, indicating the critical nature of the first few days of patient care. Importantly, the overall mortality rate on day 5 and beyond surpasses the survival rate, underlining the significance of early interventions in influencing patient outcomes. These observations underscore the potential of the DRR to serve as an early indicator of disease severity and subsequent prognosis, aiding in the timely identification of patients at higher risk of adverse outcomes.

The diagnostic accuracy of the DRR on various assessment days provides valuable insights into its predictive utility. On day 0, the sensitivity of the DRR is remarkably high, accurately identifying almost all patients who eventually succumb to the disease. However, the specificity and negative predictive value on day 0 are both quite low, indicating a higher likelihood of false positive predictions. This suggests that while the DRR might be effective in identifying patients at risk, it may not necessarily be as effective in ruling out patients who will survive.

As the assessment days progress, the specificity and negative predictive value of the DRR increase, albeit with some compromise in sensitivity. This shift suggests that the DRR becomes more effective in identifying patients who are likely to survive, particularly after day 3 of hospitalization. These findings align with the clinical intuition that the trajectory of the DRR might offer valuable insights into the evolution of disease severity and the potential for recovery.

In his research, Su et al. highlighted the significance of evaluating a secondary DRR between days 3 and 7 following ICU admission for trauma patients. Incorporating the DRR value from this period substantially improved the accuracy of all the prognosis prediction models studied [9]. Our research mirrored these findings; the DRR values at admission and on the second day post admission did not show significant differences between ICU survivors and non-survivors. However, by day 5, the DRR value noticeably increased among those who did not survive.

This phenomenon is analogous to the trajectories seen in COVID-19 cases, where the liver often becomes the secondary organ affected after the lungs. Liver damage indicators in severely affected patients include decreased platelet counts, heightened neutrophil counts, activated fibrinolytic and coagulation pathways, and increased ferritin levels [14]. These observations align with the data from a study by Kumari et al. (2023), further strengthening the link between liver injury and disease progression [10].

However, an essential observation to consider is the notable discrepancy in the cut-off value of elevated DRR across various studies on hospitalized COVID-19 patients [11,15]. It implies that while DRR holds promise, there is variability in its threshold for predicting outcomes. Such variations might be due to the nature of the study cohort; ICU-admitted patients tend to have more severe manifestations than those in general wards.

Drácz et al. found that a DRR value of ≥1.218 was notably linked with increased mortality and the severity of the disease among COVID-19 patients admitted to the hospital [11]. In a separate study by Lu et al., it was revealed that DRR exceeding 1.49 at the time of hospital admission was significantly related to in-hospital deaths in COVID-19 patients [15]. Yet, none of these investigations explored DRR values in ICU-admitted patient cohorts. In contrast, our research identified that a DRR of more than 1 at day 5 post ICU admission yielded an AUROC (95% CI) of 0.76 (CI: 0.665-0.849). This demonstrated a balanced sensitivity and specificity, at 81.82% and 57.14%, respectively, in forecasting the mortality of COVID-19 patients in the ICU. The lower cut-off in our study compared to others might be attributed to our focus on critically ill ICU patients, while the others examined general hospital-admitted COVID-19 patients.

Moreover, while the secondary DRR assessed between days 3 and 7 has proven vital for ICU-admitted trauma patients [9], it has not been evaluated in severe ICU-admitted COVID-19 patients before. Our research pioneers in emphasizing the relevance of DRR in this specific group of ICU patients, revealing a significant difference in DRR values estimated a few days post admission between survivors and those who succumbed.

The results of the logistic regression analysis further validate the prognostic significance of the DRR. The negative coefficients for both gender and DRR on day 5 indicate that both factors are independently associated with a decreased likelihood of positive survival outcomes. Males exhibit lower odds of survival compared to females, while higher DRR on day 5 is associated with reduced odds of survival. This analysis provides a deeper understanding of how the DRR, in conjunction with gender, contributes to predicting patient outcomes, enhancing the potential for personalized risk stratification.

It is important to acknowledge the limitations of this study. Being a retrospective analysis, there might be inherent biases related to data collection and incomplete medical records. Additionally, the study is limited to a single tertiary care hospital in India during a specific time frame, potentially affecting the generalizability of the findings to broader populations and settings. Future research efforts could encompass larger, multicenter studies that consider a wider range of clinical and laboratory parameters to further refine prognostic models.

## Conclusions

This study provides valuable insights into the prognostic value of the DRR in predicting survival outcomes for ICU-admitted COVID-19 patients during the second wave of the pandemic. The dynamic changes in sensitivity, specificity, and predictive values across different assessment days emphasize the evolving nature of the DRR's predictive utility. The results underscore the potential of the DRR as an early marker for disease severity, aiding clinicians in identifying high-risk patients and optimizing critical care management strategies.
